# A new type of cell related to organ movement for selfing in plants

**DOI:** 10.1093/nsr/nwad208

**Published:** 2023-08-10

**Authors:** Yin-Zheng Wang, Yan-Xiang Lin, Qi Liu, Jing Liu, Spencer C H Barrett

**Affiliations:** State Key Laboratory of Systematic and Evolutionary Botany, Institute of Botany, Chinese Academy of Sciences, Beijing 100093, China; China National Botanical Garden, Beijing 100093, China; College of Life Sciences, University of Chinese Academy of Sciences, Beijing 100049, China; State Key Laboratory of Systematic and Evolutionary Botany, Institute of Botany, Chinese Academy of Sciences, Beijing 100093, China; China National Botanical Garden, Beijing 100093, China; College of Life Sciences, University of Chinese Academy of Sciences, Beijing 100049, China; College of Pharmacy, Fujian University of Traditional Chinese Medicine, Fuzhou 350122, China; State Key Laboratory of Systematic and Evolutionary Botany, Institute of Botany, Chinese Academy of Sciences, Beijing 100093, China; China National Botanical Garden, Beijing 100093, China; College of Life Sciences, University of Chinese Academy of Sciences, Beijing 100049, China; State Key Laboratory of Systematic and Evolutionary Botany, Institute of Botany, Chinese Academy of Sciences, Beijing 100093, China; China National Botanical Garden, Beijing 100093, China; College of Life Sciences, University of Chinese Academy of Sciences, Beijing 100049, China; Department of Ecology and Evolutionary Biology, University of Toronto, Toronto, Ontario M5S 3B2, Canada

**Keywords:** stigma movement, rough endoplasmic reticulum, contractile cells, circadian moisture changes, reproductive transition

## Abstract

Many plants employ osmotic and hydrostatic pressure to generate movement for survival, but little is known about the cellular mechanisms involved. Here, we report a new cell type in angiosperms termed ‘contractile cells’ in the stigmas of the flowering plant *Chirita pumila* with a much-expanded rough endoplasmic reticulum (RER). Cryo-scanning electron microscopy and transmission electron microscopy analyses revealed that the RER is continuously distributed throughout the entirety of cells, confirmed by endoplasmic reticulum (ER)-specific fluorescent labeling, and is distinct from the common feature of plant ER. The RER is water-sensitive and extremely elongated with water absorption. We show that the contractile cells drive circadian stigma closing–bending movements in response to day-to-night moisture changes. RNA-seq analyses demonstrated that contractile cells have distinct molecular components. Furthermore, multiple microstructural changes in stigma movements convert an anti-selfing structure into a device promoting selfing—a unique cellular mechanism of reproductive adaptation for uncertain pollination environments.

## INTRODUCTION

Even though plants are essentially sessile in nature, they usually conduct reversible shape-shifting activities of organ movement over a wide range of sizes and timescales [1]. Animals usually employ contractile muscles to change shape and generate movement. However, the primary force driving plant movement is hydrostatic pressure, i.e. a force that pushes [2]. Plant movement is often associated with motor cells that expand (or contract) upon osmotic changes to generate movement [3].

A well-known example of unicellular motor cells is the guard cells of stomata that reversibly change their shape in response to various stimuli [4]. The organs that move are connected to specialized motor organs with deformation generating the organ movement, as in the case of the pulvinus in *Mimosa pudica* [3,5]. In the pulvinus, the distinct turgor pressure between motor cells on the adaxial versus abaxial sides upon a stimulus leads to a bending deformation and the resulting dynamic movement of the attached leaflets [6]. The rapid closure of the Venus flytrap (*Dionaea muscipula*), called ‘one of the most wonderful in the world’ by Darwin [1], is a consequence of the rapid release of stored energy that has accumulated due to the hydrostatic pressure difference between hydraulic layers of the lobe. The trigger signal causes fluid to rush from the inner to the outer layers with simultaneous expansion of the outer layer and shrinkage of the inner layer. This process involves asymmetric function of motor cells in the midrib and cell-wall mechanics upon triggering [2,7,8]. The changes in motor cell volume and turgor pressure through water flow are accompanied by Ca^2+^ dynamics and rapid ion influx via specific channels [9,10].

Bulliform cells are enlarged and highly vacuolated bulky cells longitudinally arranged in fan-shaped strips between vascular bundles on the adaxial epidermis of the leaf. The cells play a key role in leaf movements, i.e. leaf rolling, via turgor changes upon the loss and absorption of water [11,12]. As for hygroscopic movement, the bilayered structure of organs with a resistance tissue juxtaposed to an active tissue usually converts local swelling and shrinking into a global bending movement in response to humidity changes [1], such as the repeated opening motion of the pine-cone scales during seed release [13] and the periodic bending movement of wheat awns facilitating the penetration of seeds into soil, promoting germination [14,15]. In contrast to turgor-mediated movement activated reversibly without involving growth, the most ubiquitous movements in plants are associated with growth and are irreversible, such as the phototropic movements or solar tracking as in the sunflower (*Helianthus annuus*) [1]. Solar-tracking rhythms of young sunflowers are generated by the coordinated regulation of auxin signaling and the circadian clock on the opposite sides of the young stem [16].

Thus, great advances have been made in uncovering the mechanisms underlying plant movements over the past two decades. Nevertheless, this research has mainly involved understanding the biomechanics, physics and chemistry of plant movement. Many questions with respect to cellular and molecular mechanisms governing plant movement remain today and the field represents a treasure trove of unsolved mysteries [6].

Here, we report a new type of cell in plants associated with plant movement. We refer to these cells as contractile cells that are characterized by much-expanded rough endoplasmic reticulum (RER) with the nucleus pressed to the edge. The cells extend along the longitudinal axis of the stigma laminae in the angiosperm *Chirita pumila*. The RER water sensitivity causes cells to elongate and contract upon absorption and loss of water, which bring about a stigma circadian rhythmic movement in response to day-to-night humidity changes. Significantly, the repeated closing–bending stigma movements are of functional significance for plant sexual reproduction as they transform a typical animal-pollinated flower into a floral system that effectively promotes autonomous selfing—a novel proximal mechanism causing self-fertilization in angiosperms.

## RESULTS

### Stigma movement with humidity change

**Figure 1.**
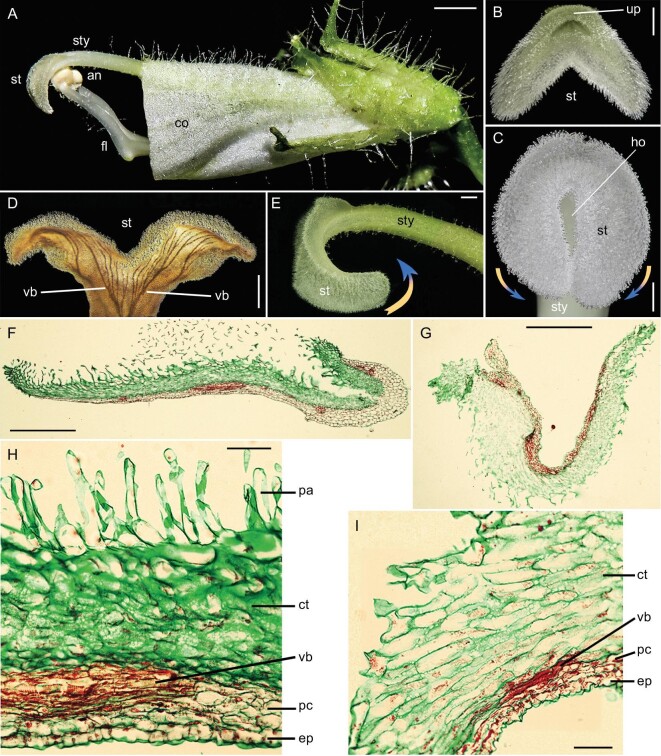
Comparison of stigma laminae at open and closed states in *Chirita pumila*. (A) The dissected flower bud with the upper part of the corolla (co) removed, showing somewhat curved stigma laminae (st), filaments (fl) geniculate at the midpoint, lifting up the pairs of coherent anthers (an) located between the style (sty) and abaxial unreceptive surface of the stigma laminae. (B) Stigma in front view, showing the arrested upper part (up) and an expanded lower part divided laterally into two oblong laminae (st) opened with an angle of 60º. (C) Closed stigma in front view with the laminae tips in contact with each other with an inter-lamina hole (ho). (D) Transparent stigma in abaxial view, showing vascular bundles (vb) distributed as a finger-like branch in each lamina. (E) Closed stigma in lateral view with the laminae strongly bent upwards against the style. Arrows exhibit the movement direction of stigma laminae. (F and G) Longitudinal anatomical sections of the (F) open/straight lamina and (G) closed/bent lamina. (H and I) Magnification of partial sections of (H) open/straight lamina and (I) closed/bent lamina, showing five types of cells from the top down, i.e. papilla cells (pa), contractile cells (ct), vascular bundles (vb), parenchyma cells (pc) and abaxial epidermis (ep). Scale bars, 2.5 mm (A), 0.5 mm (B and D), 1.0 mm (C and E), 500 μm (F and G), 50 μm (H and I).

*Chirita pumila* (Gesneriaceae) is an emerging model system for studies of floral organ structure and evolution in flowering plants [17]. The stigmas of *C. pumila* primarily consists of an arrested upper part that is almost invisible and a greatly expanded lower part that is further divided laterally into two oblong and slightly curved stigma laminae opening at an angle of 60^º^–90^º^—a state we refer to as ‘open’ (Fig. 1A, B and D) [18,19]. The laminae are usually inclined outwards 5°–10° down. In climate-controlled growth rooms, we noticed that the stigma laminae usually move repeatedly from open to closed with upward bending and vice versa following humidity changes (Fig. 1C and E). To test the causal relationship between stigma movement and humidity, we conducted a water-sensitive experiment. When we sprayed water mist over the stigma, or put a drop of water onto the surfaces of stigma laminae, we observed rapid lateral closure and upward bending of the stigma laminae after which time the laminae gradually returned to their original open state (Supplementary Videos S1 and S2). In an experiment simulating natural variation in humidity with constant temperature, when we increased the moisture in a semi-closed room with a humidifier, the stigma laminae moved from open to closed and returned as moisture decreased (Supplementary Video S3). These experiments demonstrated that the stigma laminae of *C. pumila* are water-sensitive.

### Multilayered structure for stigma movement

To address what cellular mechanisms might underlie the water-sensitive stigma movement, we conducted an anatomical investigation of the stigma laminae. In longitudinal sections from the top down, the lamina consists of papilla cells, parenchyma cells with vascular systems imbedded within them and an abaxial epidermis (Fig. 1F–I). Significantly, 8–10 layers of parenchyma cells above vascular bundles, occupying about half of the lamina thickness, exhibit a striking morphological difference between the open and closed lamina. Thus, we term them contractile cells to distinguish them from the general parenchyma cells. Then, the stigma lamina consists of five types of cells from the top down, i.e. papilla cells, contractile cells, vascular system, parenchyma cells and an abaxial epidermis (Fig. 1H). The other cell layers are similar in shape between two states with parenchyma cells (most below vascular bundles) under pressure in the closed lamina. In the open and straight lamina, the contractile cells are homogeneous with an oblong shape arranged slightly obliquely to the lamina longitudinal axis, and are compact and adherent to one another with a deep stain. But, in contrast, the cells are greatly expanded in a longitudinal direction and arranged loosely with a shallow stain in the closed and bent lamina (Fig. 1H and I).

In the contractile cells, our measurements indicate that the average cell length is 36.0 ± 0.8 μm (*N* = 20) in the open lamina but 86.5 ± 4.9 μm (*N* = 20) in the closed lamina (Supplementary Fig. S1A) (*P* < 0.001, Supplementary Data S1). We further conducted measurements of the color depth and cytoplasm density of the contractile cells. The mean OD (optical density) value is 0.5 ± 0.004 (*N* = 10) in the open lamina and 0.4 ± 0.003 (*N* = 10) in the closed lamina (Supplementary Fig. S1B) (*P*<0.001, Supplementary Data S2). There was a significant negative correlation between cell length and OD value in the contractile cells. These findings suggest that the expansion in length of contractile cells relies on the intracellular pressure that may be caused by direct water absorption by vacuoles in the cells or by some swelling material filling in the cells.

### Subcellular and statistical analyses

**Figure 2.**
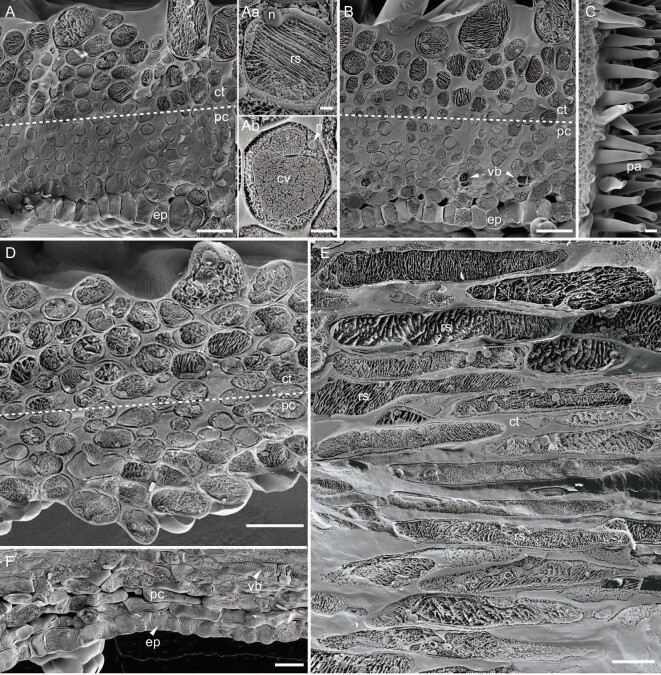
Comparison of contractile and parenchyma cells of *Chirita pumila* through Cryo-SEM. (A and B) Cross sections of contractile cells (ct) and parenchyma cells (pc) of the (A) open and (B) closed lamina. Magnification of cells from (A) showing contractile cells filled with reticular structures (rs) with the nucleus (n) pressed to the edge (Aa) and parenchyma cells having large central vacuoles (cv) (Ab). (C) Surface of stigma lamina with a large number of papilla cells (pa). (D) Longitudinal section, showing morphology of contractile cells (ct) and parenchymal cells (pc) in open lamina. (E) Longitudinal section of contractile cells (ct) significantly elongated to long cylindrical shape in closed lamina. The contractile cells are filled with reticular structures (rs). Note that (E) is a collection of images from different sections at the same scale. (F) Longitudinal section of parenchymal cells (pc) with vascular bundles (vb) and cutinized abaxial epidermis (ep). Scale bars, 30 μm (A)–(F), 3 μm (Aa) and (Ab).

To investigate these hypotheses, we analysed the cell structure of the laminae by employing cryo-scanning electron microscopy (Cryo-SEM) to examine the possible mechanism causing stigma movement. Our cross sections revealed that the contractile cells are filled with reticulate structures transversely interconnected across the full diameter of cells with the nucleus pressed to the edge (Fig. 2A and Aa). In contrast, the parenchyma cells usually possessed a large central vacuole as frequently observed in plant parenchymal tissues (Fig. 2A and Ab). Both contractile and parenchyma cells were only slightly widened after water absorption (Fig. 2A and B). In longitudinal sections, however, we found that the contractile cells were significantly elongated from an elliptical or spherical to an extremely long cylindrical shape after water absorption whereas parenchyma cells were almost unaffected by moisture change (Fig. 2D–F). The papilla cells are located above the contractile cells in a perpendicular orientation on the laminar surface (Fig. 2C). This finding implies that the vacuoles may not account for the significant elongation of contractile cells after water absorption.

**Figure 3.**
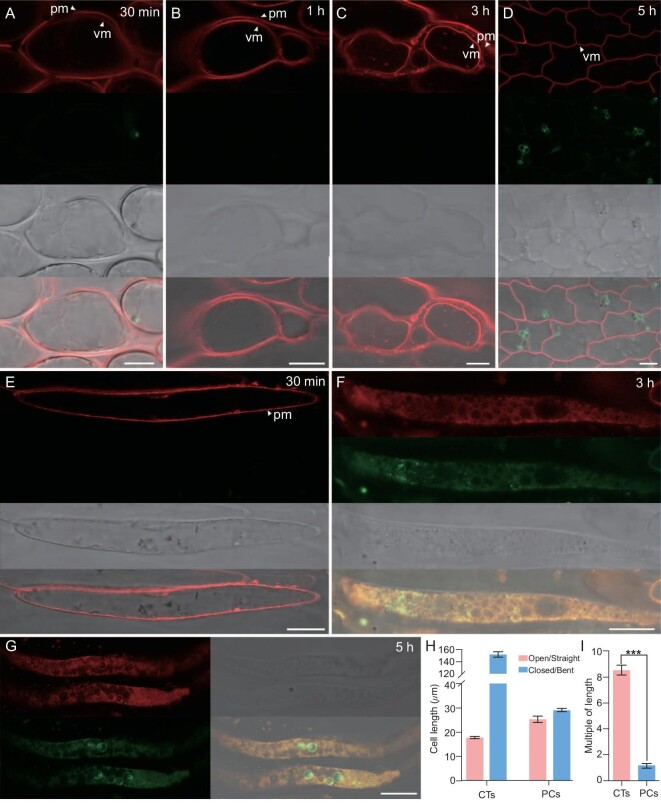
Confocal analysis of parenchyma and contractile cells in *Chirita pumila* labeled with FM4-64. At each time period, the signals of wavelengths of 580–630 nm (red), 480–520 nm (green), bright field channels and merge images are arranged from top to bottom. (A–D) Transition of FM4-64 fluorescence signals in parenchyma cells at different chasing times. FM4-64 signals are mainly located in the plasma membrane (pm) at 30 min (A), equally bright in plasma membrane (pm) and vacuolar membrane (vm) at 1 h (B) and specifically targeted at the vacuolar membrane (vm) from (C) 3 h to (D) 5 h. (E–G) Changes in FM4-64 signals in contractile cells at different chasing times, showing FM4-64 signals only observed in plasma membrane (pm) at 30 min (E) and then disappeared after 3 h (F and G). Meanwhile, the green non-VM-specific signals (wavelength of 480–520 nm) emerge in the cells after 3 h (F and G). (H) Average cell lengths of contractile cells (CTs) and parenchyma cells (PCs) in the open/straight and closed/bent laminae. Values shown are mean ± standard error of the mean, *P*<0.001. (I) Statistical results of the multiple of cell-length changes. Error bars represent standard deviation. ***P ˂ 0.001. Scale bars, 10 μm (A)–(D), 20 μm (E–G).

To confirm this result, we employed confocal laser scanning microscopy (CLSM) to visualize the vacuoles and related compartments in the stigma laminar cells with FM4-64. FM4-64 is a fluorescent dye that usually serves as a robust marker of the plant vacuolar membrane (VM) but does not label the endoplasmic reticulum (ER) and the nuclear envelope, or does so much less efficiently than the VM [20]. Since FM4-64 dye is usually gradually transferred from the plasma membrane to the VM, we stained cells for a limited time and then chased for several hours. In addition, a short-wavelength (480–520 nm) excitation was used to visualize non-VM-specific signals in comparison with the VM-specific signals. Soon after the laminar cells were stained with FM4-64, the dye became localized to the plasma membrane in both the contractile and parenchyma cells (Fig. 3A and E). Afterwards, FM4-64 fluorescence signals in the parenchyma cells became equally bright in the plasma and vacuole membranes at 1 h (Fig. 3B) and then gradually disappeared from the plasma membrane and became specifically targeted to the vacuole membrane after 3 h (Fig. 3C and D). In contractile cells, FM4-64 signals completely disappeared from the plasma membrane after 2 h, but no VM-specific signal was detected afterwards (Fig. 3F and G). In addition, weak red signals co-localized to green signals of short wavelength after 3 h displaying a longitudinally expanded reticulate structure fully filling the contractile cells (Fig. 3F and G). These results showed that parenchyma cells frequently hold one large central or two separate vacuoles while contractile cells have no vacuole but a reticulate structure.

We further conducted measurements of cell length in the lamina between open and closed stages with comparison between contractile and parenchyma cells. In parenchyma cells, the average cell length is 25.363 ± 0.333 μm (*N* = 84) in the open lamina and 29.218 ± 0.354 μm (*N* = 84) in the closed lamina (Fig. 3H). In contractile cells, the average cell length is 17.870 ± 0.115 μm (*N* = 51) in the open lamina but 152.213 ± 2.270 μm (*N* = 84) in the closed lamina (Fig. 3H) (*P* < 0.001, Supplementary Data S3). From the open to closed stages in contractile cells, the cell length increased by >8-fold (8.52 ± 0.072) but was almost unchanged in parenchyma cells (1.15 ± 0.018) (Fig. 3I) (*P* < 0.001, Supplementary Data S4), indicating that the vacuoles did not significantly expand due to water absorption. In contractile cells, the cell length is in fact the length of the reticulate bodies because the cells were fully filled with reticulate bodies transversely and longitudinally, occupying almost all the space of these cells with the nucleus pressed to the edge. Apparently, the change in length of reticulate bodies is strongly correlated with the deformation of stigma laminae from the open to the closed stages. These findings suggest that the intracellular pressure driving great elongation of contractile cells is not caused by vacuoles. Instead, this change is produced by the swelling materials, i.e. the reticulate bodies, that fill contractile cells. The extreme elongation of the reticulate bodies with water absorption are therefore the driving force for the deformation of stigma laminae causing the open-to-closed stigma movement.

### Ultrastructure of reticulate bodies and organelle identification

**Figure 4.**
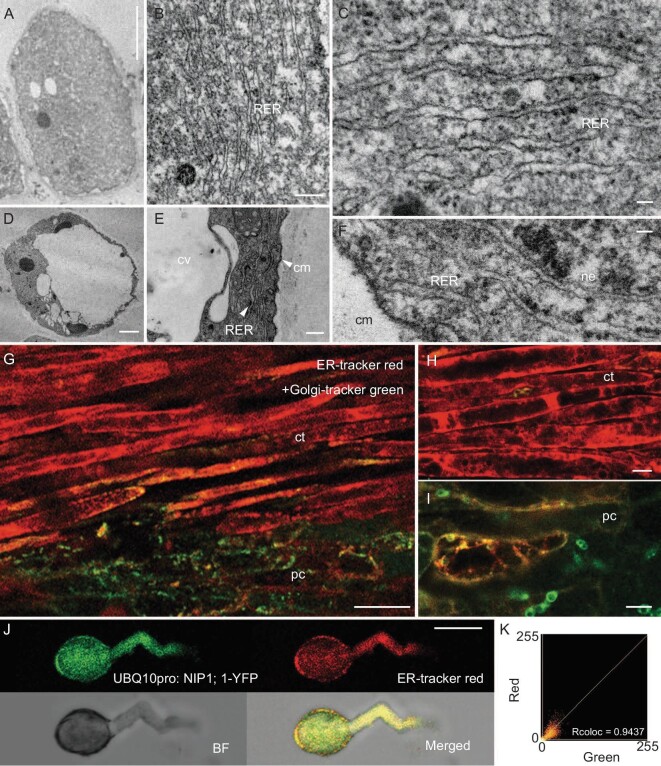
Analyses of contractile and parenchyma cells in *Chirita pumila* by using TEM and fluorescent labeling. (A)–(C) TEM images of contractile cells having no obvious vacuolization (A), but filled with abundant rough endoplasmic reticulum (RER) evidently studded with ribosomes at the surface (B and C). (D–F) TEM images of parenchyma cells having large central vacuoles (cv) (D) with small amounts of RER distributed only at the cell periphery (E and F). (G–I) Confocal images of fresh stigmas stained by using ER-Tracker Red and Golgi-Tracker Green dyes with ER-Tracker fluorescence signals in contractile cells (ct) and Golgi-Tracker in parenchyma cells (pc) (G); (H) magnified contractile cells show strong reticular ER-Tracker signals and (I) parenchyma cells show obvious Golgi-Tracker signals. cm, cytomembrane; ne, nuclear envelope. (J and K) Colocalization of ER-Tracker Red with NIP1;1. (J) Colocalization analysis of ER-Tracker Red with *UBQ10pro: NIP1;1-YFP*, showing a significantly co-localized relationship with each other (Rcoloc = 0.9437). (K) *UBQ10pro: NIP1;1-YFP* and ER-Tracker Red displaying a strong colocalization. Scale bar, 2 μm (A and D), 0.2 μm (B and E), 50 nm (C and F), 50 μm (G), 10 μm (H and I), 20 μm (J).

To determine which kind of organelles the reticulate bodies are in contractile cells, we used transmission electron microscopy (TEM) to analyse contractile cells compared with parenchyma cells. At low magnification, it was further confirmed that the contractile cells have no obvious vacuolization, whereas the parenchyma cells exhibited giant vacuoles characteristic of mature plant cells (Fig. 4A and D, and Supplementary Fig. S2A and D). At high magnification, we found that the contractile cells have abundant RER evidently studded with ribosomes at the surface (Fig. 4B and C, and Supplementary Fig. S2B and C). The RER is distributed throughout the entire cell, rarely with Golgi bodies, indicating that the reticulate bodies are in fact the RER. By contrast, in parenchyma cells, we observed some Golgi bodies but only a small amount of RER arranged only along the periphery of cells (Fig. 4E and F, and Supplementary Fig. S2E and F).

To confirm this finding, we further employed CLSM to observe *in situ* live-cell florescence signals with probe dyes of ER-Tracker Red specific for the ER and Golgi-Tracker Green for Golgi bodies, respectively. Our results revealed that the contractile cells have strong signals of reticulate ER-Tracker Red with almost no specific Golgi signals, whereas Golgi-Tracker Green signals are easily observed in parenchyma cells without obvious ER-Tracker Red signals (Fig. 4G–I). We then conducted an experiment on the colocalization of ER-Tracker Red and the ER marker NIP1;1 in fusion with a yellow fluorescent protein (YFP) (YFP: NIP1;1) in the pollen tube of transgenic *Arabidopsis thaliana*. This experiment confirmed that the ER-Tracker Red probe is specific to the endoplasmic reticulum. Our results provide strong evidence for the nature of the reticulate structure, i.e. the RER (Fig. 4J and K).

As outlined above, the great elongation of the contractile cells could not be caused by expansion of vacuoles with water absorption because of the lack of vacuoles in the contractile cells and, besides, no significant expansion of vacuoles occurred in the parenchyma cells with water absorption (Figs 2Aa and F and 3). The extreme elongation of the contractile cells is actually caused by the swelling materials that are in fact the RER filled in the contractile cells with a >8-fold increase in length from the open to the closed stages upon water absorption (Fig. 3H and I). Our findings suggest that the RER, acting as a swelling material, plays a key role in the rapid and significant elongation of the contractile cells with water absorption causing subsequent stigma movement in response to humidity changes.

### Function of stigma movement

*Chirita pumila* is an annual herb with showy zygomorphic flowers and, in common with other species in the genus, the flowers are adapted for cross-pollination by insects [18] (Supplementary Fig. S3). However, our previous studies revealed that *C. pumila* most often self-pollinates in the bud prior to anthesis, i.e. it exhibits pre-anthesis cleistogamy—an exception in comparison with other *Chirita* species [19] (Supplementary Fig. S4). In addition to a suite of floral traits associated with insect-mediated cross-pollination, the anthers and stigma are arranged in an elaborate structure that specifically avoids autonomous self-pollination. In this structure, two uplifted face-to-face cohered anthers are located between the style and abaxial surfaces of the curved stigma laminae, which makes the downward adaxial receptive surface remote from the anthers—a typical anti-selfing structure [19] (Fig. 1 and Supplementary Fig. S3).

**Figure 5.**
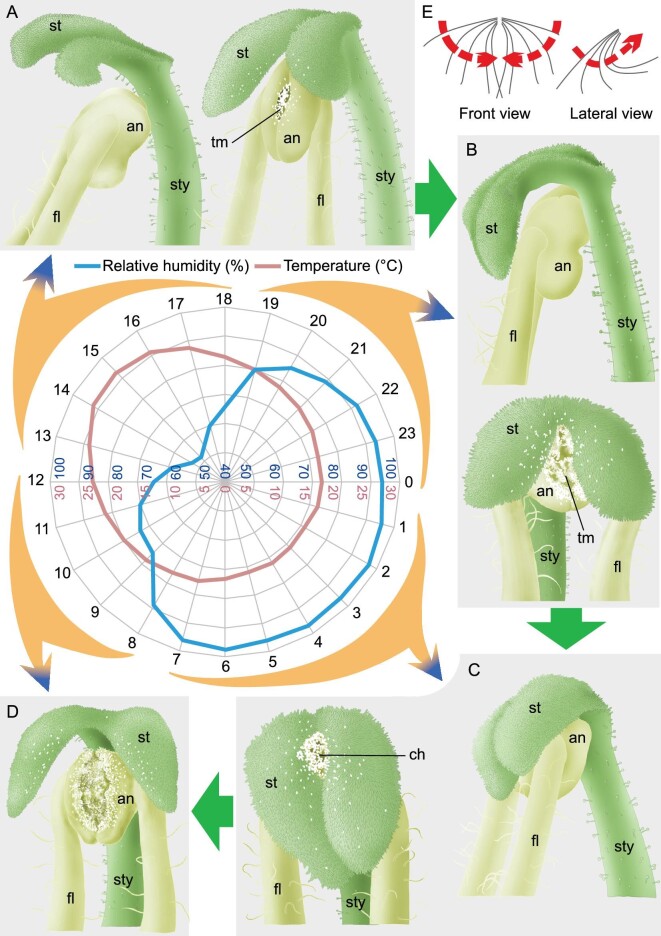
Stigma movement in *Chirita pumila* and relative humidity and temperature variation observed and recorded under field conditions (drawn in Supplementary Fig. S5). (A) Stigma laminae (st) at open state in the afternoon. (B) Stigma laminae gradually closing from early evening to night. (C) Stigma laminae closed from midnight to dawn with lamina tips overlapped, mechanically squeezing anthers (an) against the style (sty). (D) Stigma laminae returned to the open state from morning to noon. The direction to the style is upward and the opposite downward. The numbers covered by large arrows that point to related images indicates the time period (Beijing time, UTC + 8) corresponding to the state of stigma movement as shown in the images in a circadian cycle (from 13–18, 19–0, 1–7 to 8–12). Note: an initial rhombic dehiscent stomium (tm) at the juncture of the face-to-face cohered anthers (an) (A) that is extended to the full length of the coherent anthers (B), and a pollen channel (ch) formed by stigma movement, making the stomium located just below the inter-lamina hole with movement to closed (C). (E) Bidirectional movement paths of the stigma laminae, laterally closing (front view) and upwards bending (lateral view). The center portion shows average daily variation of relative humidity (blue lines) and temperature (pink lines) at a site in which *C. pumila* occurs naturally in Hekou county, Yunnan province, China.

To examine whether stigma movement was functionally related to self-pollination in the bud, we conducted continuous observations for 24-h time periods in natural populations in the wild. In the afternoon, the stigma laminae were open, as described above (Figs 1 and 5, and Supplementary Fig. S5). Later, they gradually closed and bent. At dawn, the two stigma laminae were completely closed laterally with lamina tips in contact with each other and strongly bent upwards against the style, mechanically squeezing the anthers (Fig. 5, Supplementary Fig. S5 and Supplementary Video S4), but then gradually returning to the open and straight position, following a day-to-night pattern. Our field measurements recorded that the average relative humidity during 7 days at one site varied from ∼97% at dawn to 52% in the afternoon (Fig. 5, Supplementary Fig. S5 and Supplementary Table S1). These values are consistent with the official records of a weather station near the site with average relative humidity varying from ∼90% to 60% (Supplementary Fig. S6 and Supplementary Data S5). Apparently, under natural conditions, the stigma laminae exhibit a circadian rhythmic movement associated with humidity change. This finding agrees with the data observed in the greenhouse, where only humidity was changed (Supplementary Video S3).

We further explored whether there is any possible structural intermediary that might cause pollen transfer from anthers to stigmas. We observed that, in the closed bud prior to anthesis, pollen grains mature precociously accompanied by a rhombic dehiscent stomium initially formed at the ventral juncture of two face-to-face cohered anthers. The stomium further extends to full length along the ventral side of the coherent anthers (Fig. 5A and B, Supplementary Fig. S5A and B, and S7). In addition, the stigma movement is bidirectional, closing laterally and bending upwards, as the laminae are inclined outward 5°–10° down. On closure, the stigma leaves a hole between two closed laminae and the dehiscent stomium is located just below the inter-lamina hole as a pollen channel (Figs 1C and 5B and C, Supplementary Fig. S5B and C, and Supplementary Video S5). We observed that there was a considerable number of pollen grains that emerged around the inter-lamina hole on the surface of the laminae when the stigma laminae became closed and bent. As the stigma laminae returned to an open and straight position, we observed that the anthers become squashed or pressed out of shape with a large amount of pollen extruded from the inside of the anthers (Fig. 5C and D, and Supplementary Fig. S5C and D). After several rounds of open-to-closed stigma movement, the anthers were almost empty of pollen and completely opened (Fig. 5D, Supplementary Fig. S5D and Supplementary Video S5). According to these observations, pollen grains are most likely forcibly ejected from the pollen channel as the stigma laminae continuously exert mechanical pressure on the anthers and fall onto the stigma receptive surface causing self-pollination because the stigmas and anthers synchronously mature precociously (Fig. 5C, Supplementary Figs S4, S5C and S7, and Supplementary Video S6). These series of events are synchronized with mechanical squeezing of the anthers by the stigma, thus forcing pollen grains to be ejected from the channel and falling onto the receptive stigma surface. Therefore, the cellular processes governing changes in organ position function in transforming a classic anti-selfing structure adapted for cross-pollination to a device specifically for promoting autonomous selfing.

### Comparative RNA-seq analyses

**Figure 6.**
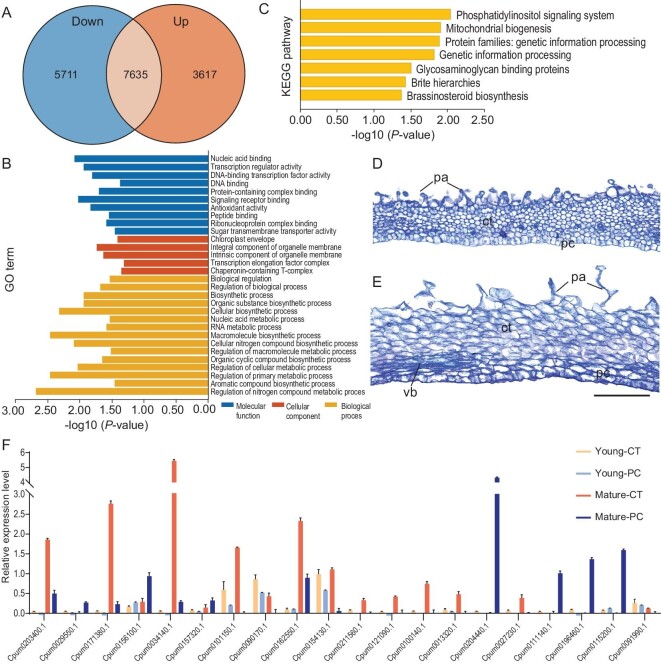
Comparative RNA-seq analyses of contractile and parenchyma cells in *Chirita pumila*. (A) Venn diagram of the number of DEGs in contractile cells (CTs) and parenchyma cells (PCs). (B) GO enrichment analysis in DEGs specific for CTs with DEGs significantly enriched into three classes (*P* ≤ 0.05), i.e. cellular component, molecular function and biological processes. The figure shows the top GO terms for the number of genes in each class. (C) KEGG enrichment analysis in DEGs specific for CTs with DEGs significantly enriched into seven pathways (*P* ≤ 0.05). (D) Longitudinal anatomical sections of the open/straight lamina in young stage (length of flower buds < 0.5 cm). No differentiation observed between CTs and PCs with uniform small rounded cells in young stigmas. (E) Longitudinal anatomical sections of the open/straight lamina in mature stage (length flower buds ≈1.5 cm). The oblong CTs were arranged slightly obliquely to the lamina longitudinal axis while elliptic or oblong PCs were arranged in line along the lamina longitudinal axis. Scale bar, 100 μm (D and E). (F) Real-time PCR validation of 20 representative DEGs identified in the transcriptome. AP2/ERF, *Cpum0121090.1*; B3 Domain, *Cpum0013320.1*; bZIP, *Cpum0115200.1*; PLATZ, *Cpum0027230.1*; NAM, *Cpum0211560.1*; HSF, *Cpum0091990.1; Cpum0111140.1*; bHLH, *Cpum0196460.1*; WRKY, *Cpum0100140.1*; GATA, *Cpum0101150.1*; MYB, *Cpum0204440.1*; RTNs, *Cpum0090170.1, Cpum0162550.1*; NET, *Cpum0156100.1, Cpum0034140.1, Cpum0157320.1*; FH, *Cpum0203400.1, Cpum0029550.1, Cpum0171380.1*; and RHD3, *Cpum0154130.1*. The sequence of representative DEGs and the primers used for real-time PCR are given in Supplementary Data S7.

To determine whether there was significant molecular differentiation between contractile and parenchyma cells, we collected the cell types by using laser microdissection (Supplementary Fig. S8) and performed comparative RNA-seq analyses. We identified 9328 differentially expressed genes (DEGs), with 3617 and 5711 genes up- and downregulated in contractile cells, respectively (Fig. 6A), implying a differential role of the contractile cells from parenchyma cells. In Gene Ontology (GO) and Kyoto Encyclopedia of Genes and Genomes (KEGG) enrichment analyses, DEGs specific for contractile cells were significantly enriched into three classes and seven pathways, respectively (*P* < 0.05) (Fig. 6B and C, and Supplementary Data S6). We identified a large number of DEGs in contractile cells that are involved in the dynamics of ER, membrane signaling, immune responses and stress resistance, consistently with the morphology of contractile cells filled with abundant RER. The DEGs significantly enriched for up- or downregulation were from AP2/ERF, B3-Domain, bZIP, PLATZ, NAM, heat shock factor (HSF), bHLH, WRKY, GATA and MYB transcription factors, and Reticulons (RTNs), Networked (NET), Formin (FH) and ROOT HAIR DEFECTIVE3 (RHD3) protein families. Using real-time PCR, we further examined the expression of 20 representative genes in young and mature stigmas (Supplementary Fig. S8). Their expression differentiations were weak in young stigmas but very significant in mature stigmas between the two cell types, which correlated with their undifferentiation of small rounded cells in young and striking differentiation in mature stigmas, with contractile cells derivatively arranged obliquely to the lamina longitudinal axis (Fig. 6D). Our RNA-seq analyses revealed that contractile cells have remarkably different molecular properties and components from parenchyma cells, indicative of their distinct function in the stigma laminae.

## DISCUSSION

The ER is the most extended organelle with a large reticulated and dynamic structure that serves many roles in the cell, including calcium storage, protein synthesis, lipid metabolism and also as a secretory pathway [21]. The ER is responsible for the synthesis of one-third of the cellular proteome and linked to the major auxin biosynthetic and transport activities in plants [22]. In animals, the ER is continuously distributed in a 3D network throughout the entirety of most cells [23]. In plants, however, most ER is functionally 2D and appressed between the cell wall/plasma membrane and the vacuole, which is anchored to the plasma membrane, similar to a spider web hanging from a surface [22,24,25]. Only the strands of ER, a very small part of the total volume, can cross the vacuole and connect with the nuclear envelope [22]. In contrast, our observations indicate that the RER in the contractile cells of *C. pumila* exhibits a 3D network structure that occupies almost the entire interior of the cell, with the nucleus pressed to the cell edge (Fig. 2). Apparently, the characteristics of RER in contractile cells are quite distinct from the common features of plant ER [24,25]. The ER in parenchyma cells differs in being much more consistent with common features of plant ER. Correlatively, our RNA-seq analyses revealed that the contractile cells exhibited distinctly different molecular properties and components from parenchyma cells, including RTNs, NET, FH and RHD3 proteins related to the RER architecture and dynamics [26,27].

In animals, plasma cells associated with the regulation of humoral immunity exhibit prominent amounts of fluid RER and secretory vesicles [28]. In addition, the ER tends to be greatly vesicularized during rapid responses to membrane permeabilization by cell treatments, independently of cytosolic Ca^2+^ changes [29]. Given this, we hypothesize that the RER membrane would be transformed into a mixed mucous material that expands as humidity increases because of the large amount of hydrophilic proteins and hygroscopic metabolic products localized to it. This hypothesis is consistent with our RNA-seq data, with a large number of DEGs involved in ER dynamics, membrane signaling, immune responses and stress resistances in the contractile cells. Thus, since the RER in stigma laminae is water-sensitive, it significantly expands as a rapid response to water absorption with resultant extreme elongation of contractile cells. The RER therefore seems likely to play a significant role in the stigma movement of *C. pumila* in response to humidity changes.

To the best of our knowledge, this is the first report of this specialized form of RER in plants. Because the papilla cells, a specialized thin-walled secretory tissue with high intercellular permeability, are located above the contractile cells in a perpendicular orientation on the laminar surface, they could likely absorb aerial water that directly diffuses into contractile cells by osmosis. Thus, contractile cells significantly elongate and contract upon gain and loss of water. In this process, the finger-like distributed vascular bundles and cutinized abaxial epidermis function as antagonists by exerting a resistant force against the expansion of contractile cells. This cellular interplay controls the laminae movement during moisture changes. The contractile cells with much-expanded RER are characteristic of the stigma laminae and function in initiating the stigma movement in response to changes in moisture.

As outlined in the introduction, the movement of living organs in plants is mainly attributed to motor cells, or the motor organ that is connected to an organ that moves, but is generally undeformed except for the unicellular motor cell in guard cells. In *M. pudica*, the opening and closing movements of the leaflets are driven by the rapid bending deformation of the motor organ pulvinus upon osmotic and hydrostatic pressure gradients but with the leaflets themselves undeformed [3]. The fan-shaped bulliform cells, also called motor cells, are connected to other regions of the leaf at cross section. Under drought conditions, the bulliform cells lose turgor pressure and shrink, which causes leaf rolling—an inward movement of other leaf regions that are generally unchanged in shape but moved [11,12]. These movement organs usually comprise two closely connected components, i.e. the motor organ and the motion organ that is movable and can be watched by eye but is in fact unchanged in shape itself [3,5]. Therefore, these plant movements often involve only partial deformation of organs, i.e. the activities of motor cells with resultant asymmetric deformation of the motor organ. In general, these types of plant movements look like the opening and closing of an automatic door with the door plank driven by the door axis controlled by an automatic motor machine. Movements involving deformation of whole organs are often seen in hygroscopic movements—a type of passive movement relying on changes in the water content of dead tissues and often employed in plant seed dispersal, such as the repeated opening motion of the pine-cone scales [15].

In animals, movements usually involve deformation of the motion organs or whole bodies with coupling of excitation–contraction [30]. In invertebrates, their bodies rely on a hydroskeleton for support and movement that involves deformation of motion organs or whole bodies with the force transmitted through internal liquid pressure. The incompressible liquid is enclosed in a flexible cylindrical cavity or a series of flexible segmental cavities surrounded by muscles and connective tissue fibers that allow the body to be smoothly bent [31]. In mammals, the body or organ movements are mainly powered by contraction of muscles that consist of bundles of contractile cells elongated and fully extended along the longitudinal axis of each organ of the body. In this case, movements involve deformation of motion organs or whole bodies. Interestingly, the muscle cells contain abundant sarcoplasmic reticulum—a specialized form of the smooth ER that is transversely interconnected across the full diameter of the muscle fiber that extends the length of striated muscle cells [30,32].

In *C. pumila*, the contractile cells contain abundant RER distributed throughout the entire cell. The contractile cells are fully extended along the longitudinal axis of stigma laminae, occupying about half of the lamina size, and are parallel to the vascular bundles and cutinized abaxial epidermis. Therefore, the stigma movement occurs by deformation of the whole stigma laminae as contractile cells elongate and contract with changes in osmotic pressure in response to moisture alterations. The process in *Chirita* represents a novel type of plant movement and is distinct from the general features of other organ movements in plants without deformation of the motion organs themselves, as mentioned above [3,5]. Thus, the contractile cells in *C. pumila* function in a concerted manner through multiple fine-tuning during stigma movement and transform a typical animal-pollinated flower characteristic of Gesneriaceae to a floral system that effectively promotes autonomous selfing. We interpret this unique self-pollination mechanism as an adaptive evolutionary response to unsatisfactory pollination environments in which reproductive assurance is favored when high moisture levels limit pollinator activity [33].

Finally, cells in complex organisms usually have multiple functions during development. For example, in Pacific bluefin tuna (*Thunnus orientalis*), the lymphatic fluid functions in immune response and homeostasis while also playing a key role in the pressure-driven repeated movements of fins co-opted with inclinator muscles for swimming control—a movement upon hydro-osmotic effect in fish organs [34]. Given that papilla cells just above the contractile cells in *C. pumila* are aerially exposed with their cuticular and fibered walls usually significantly disrupted or even absent [35], it would be interesting to know whether a defense system develops in the stigma laminae in response to outside invading antigens. Therefore, it would be attractive to explore whether the contractile cells are also involved in other functions besides organ movements, such as immune responses and stress resistance. In addition, future work is required to clarify how this novel cell type originated, especially the molecular mechanisms and evolutionary processes underlying its origin, and whether this specialized reproductive strategy occurs in other lineages of flowering plants.

## MATERIALS AND METHODS

For field investigation, we conducted field studies of *C. pumila* at a field site during the flowering seasons of 2016, 2017 and 2019 in southeastern Yunnan province, Southwestern China (Lat. 22°54′6.1″N, long. 104°2′4.23″E, alt. 1366 m, Laowang village, Miechang Town, Maguan County). Experimental materials were from both cultivated and wild plants, depending on different purposes. We recorded relative humidity and temperature data continuously in the field using a Temperature-Humidity recorder (Elitech RC-4) from 16 to 22 October 2019.

For Cryo-SEM, samples of fresh mature stigmas were frozen and conditioned using the PP 3010T Cryo Transfer System (Quorum Technologies). Cryo-fractures were operated directly in the chamber with a cooled knife, subjected to sublimation at –90°C for 10 min, then coated and observed by using a Regulus 8100 (Hitachi, Japan) [36]. For TEM, fresh mature stigmas were fixed in 4% paraformaldehyde and 3% v/v glutaraldehyde in 0.1 M PBS (pH 7.0), then post fixed in 1% w/v OsO4 in PBS. Samples were dehydrated through an ethanol series, embedded in Spurr's resin (Sigma-Aldrich) and polymerized at 60°C for 24 h. Ultra-thin sections (70 nm) made by using a diamond knife microtome (Leica Ultracut R) were placed on 100 mesh copper grids and sequentially stained in 2% w/v uranyl acetate for 30 min, 0.2% w/v aqueous lead citrate for 5 min and examined using a JEM-1230 (JEOL, Japan) operating at 80 kV.

For CLSM, we cut fresh stigmas into the smallest possible tissues with a knife, then placed samples in the dyes with ER-Tracker Red (1 μM; Beyotime, catalog no. C1041) and Golgi-Tracker Green (0.33 g/L; Beyotime, catalog no. C1045S) for 30 min at 25°C [37]. For FM4-64, samples in the dye-complemented liquid medium (5 μM FM4-64; MedChemExpress, catalog no. HY-103466) were incubated for 20 min in darkness on an orbital shaker at 80 r/min, then washed three times and put in fresh medium to achieve vacuole-specific staining. Fluorescent signals were detected by using a Zeiss LSM980 (Carl Zeiss, Germany). Signals of ER-Tracker and Golgi-Tracker were excited at 594 and 495 nm, detected at 615 and 488 nm emission, respectively. FM4-64 signals were detected at 480–520 and 580–630 nm, respectively.

For colocalization of the ER-Tracker Red and transgenic line UBQ10pro: NIP1;1-YFP in pollen tubes, the coding sequence of *NIP1;1* with promoter of *UBIQUITIN10* was built into the vector pNIGEL 07 with YFP sequence, which was introduced into *A. thaliana* ecotype Columbia-0 by *Agrobacterium tumefaciens*-mediated transformation for transgenic expression of NIP1: YFP proteins [38,39]. The mutant was numbered CS781650 in the Arabidopsis Biological Resource Center. Fluorescence signals of YFP were excited at 505 nm, detected at 520 nm emission. Germinated pollen of the transgenic line *in vitro* was stained using ER-Tracker Red for 30 min and observed by using a Zeiss LSM980. Other methods, materials and data collection are provided in the Supplementary Data.

## Supplementary Material

nwad208_Supplemental_Files
