# Supplementary material for: A new type of cell related to organ movement for selfing in plants
Source: Natl Sci Rev. 2023 Aug 10;10(9):nwad208. doi: 10.1093/nsr/nwad208 (PMC10434738; doi:10.1093/nsr/nwad208)
Supplement: nwad208_Supplemental_Files [file nwad208_supplemental_files.zip › NSR_MS-2023-309-Manuscript-Final-Suppl Data.pdf]

Supplementary Data

A new type of cell related to organ movement for selfing in plants

Yin-Zheng Wang[1,2,3†*], Yan-Xiang Lin[1,2,3,5†], Qi Liu[1,2,3†], Jing Liu[1,2,3], Spencer C. H. Barrett[4]

[*]Corresponding author. Email: wangyz@ibcas.ac.cn

This PDF file includes:

- Materials and Methods
- Figures S1 to S8; Table S1
- Captions for Videos S1 to S6
- Captions for Data S1 to S7

Materials and Methods

Sectioning and staining

Sectioning

We cut off stigmas and these were fixed in FAA (1:1:18 v/v formalin:glacial acetic acid:50% ethanol), and vacuumed with a vacuum pump (Eppendorf Concentrator plus) for 20min, then stored at 4 ℃ for 48 h, and kept in 50% ethanol. Samples were dehydrated, cleared in an alcohol-xylene series, gradually infiltrated with paraffin at 42 ℃, and embedded in paraffin by tissue embedder (Microm EC 350-1) at 60 °C. We cut longitudinal sections, 8μm thick with steel knives using a rotary microtome (Microm HM 340 E) and these were put onto a slide dryer (Kedee KD-H) to float the slices and dry them; these were then incubated at 42 ℃ for 48h [40].

Staining

Safranin O and Fast Green staining for sectioned stigmas

The sections were deparaffinized and re-hydrated through xylene, xylene:100% ethanol (1:1) and a graded ethanol series from 100% to 50%, then stained with Safranin O (Macklin) (1%, 50% ethanol) for 13h and Fast Green (Macklin) (1%, pure ethanol) for 8s [40].

Toluidine Blue staining for sectioned anthers

The sections were deparaffinized and re-hydrated through xylene, xylene:100% ethanol (1:1) and a graded ethanol series from 100% to 0% (ultra-pure water), then stained with Toluidine Blue (Sigma-Aldrich) (0.2%, 0.1M phosphate buffer, PH=7) for 60s [40]. After being cleared in alcohol-xylene series, the slides were sealed with Neutral Balsam and examined and photographed using photomicroscope (Leica DM4000 B).

Aniline Blue staining for pollen germination on stigmas

After fixation in FAA (described above), the stigmas were kept in 50 % ethanol, and then transferred to 2mol/L sodium hydroxide solution, water bath heating at 65 ℃ for 40min, and stained in decolorized aniline blue (Macklin) (0.1%, aqueous 0.1M K_3PO_4) in a water bath at 65 ℃ for 4h. Stigmas were then squashed and sealed with glycerine [41]. We examined slides and photographed them using fluorescence microscope (Zeiss Axio Imager A1).

Tissue optical clearing for stigma

After fixation in FAA (described above), the stigmas were transferred to 2mol/L sodium hydroxide solution, water bath heating at 65 ℃ for 40min, and then kept in 50 % ethanol [19]. The transparent tissues were photographed by stereoscope (Leica S8AP0).

Photographs and videos

The images in this research were examined and photographed using a digital camera (Canon G3X) and videos were recorded by video recorder (Sony HDR-CX700) and stereoscope (Leica S8AP0).

Water sensitive experiment

We first dissected the flower bud, exposed the stigma or stigma and anthers, and then sprayed water as mist over stigmas, or put a drop of water onto the surfaces of two stigmatic laminae using a dropper or sprayer.

In the experiment simulating natural variation in humidity, we used Humidifier (Deerma DEM-F450) to control the relative humidity in a semi-closed room with consistent temperature.

Image measurement, calculation and data collection

Images measurement

In the measurement of cell length and cytoplasm density of the sections of paraffin-embedded stigmatic laminae, the images were quantified and analysed using the measurement tool in ImageJ (NIH) image analysis program (https://imagej.nih.gov/ij/index.html, public domain of the National Institutes of Health (NIH), Bethesda, MD, USA). We inputted the section images into ImageJ and converted them to 8-bit images (display 256 (2^8) gray levels), using the function Unc. OD = log 10 (255 /Pixel value) to convert gray values from 8–bit images to uncalibrated optical density values. Then, we adjusted the Threshold to guarantee Area to cover the whole upper parenchyma cell cytoplasm. The mean optical density (Integrated density/Area) were finally analyzed and measured by the ImageJ software [42]. The cell lengths were measured with the scale under the photomicroscope (Leica DM4000 B).

Calculation

We calculated the average and SEM (standard error of the mean) of cell length, as well as the average and SEM of mean optical density, by Microsoft Excel, respectively. We determined *P* values for cell length and mean optical density using *t*-tests. Relative humidity and temperature data recorded in the field and the weather station were also sorted and calculated by Microsoft Excel, respectively.

Data collection and plotting

We collected relative humidity and temperature data from real-time field records and official weather station information. The data include the 24-hour continuous records of the relative humidity and temperature of seven days at one field site of *Chirita pumila* from 16th to 22th October 2019, and relative humidity and temperature variation recorded at an official weather station near the field site of *C. pumila* from July 1st to December 31th in 2017. The field site is located at lat. 22°54'6.1"N, long. 104°2'4.23"E and alt. 1366m, Laowang village, Miechang Town, Maguan County, southeastern Yunnan province in Southwestern China. The official weather station is Mengzi weather station located at lat. 23°15'36"N, long. 103°11'24"E and alt. 1313m, Mengzi county, southeastern Yunnan province in Southwestern China. The relative humidity and temperature data were plotted in Microsoft Excel. The cell length and OD mean values were plotted in GraphPad Prism 7.

Stigmatic movement simulation

This animation was produced by the software program 3DS Max (https://www.autodesk.com/), a tool for simulation and rendering ability for 3D visualization. We created the shapes of the pistil and stamens with parameters based on the measurements of living floral organs using the software program. These objects were further organized and edited to simulate the stigmatic movement during the process of self-pollination [43].

Tissue dissociation and different type cells isolation

For cryo-section, the fresh stigmas were dissected from at least ten flowers 0.5cm-1.5cm in length and immediately embedded in OCT medium (SAKURA, Tissue-Tek) which poured on the object stage. The sample is positioned well in OCT medium for cutting and then placed on a cold table. In at least 20 minutes, OCT was completely frozen, the cryotome (Leica CM 1950) was used to cut 100μm sections [44]. The sections were washed by RNase-free PBS and carefully placed on RNase-free slides specially designed for laser microdissection (Leica, FrameSlides, POL-Membrane). All operations were completed as soon as possible at low temperature and all tools were treated to ensure RNase-free.

For laser capture microscopy, contractile cells and parenchyma cells were collected separately using a laser microdissection machine (Leica LMD7000). The slides were detectable in a 10× visual field, and we selected the target area on the computer for laser cutting into collecters

which had been previously added to RNA lysis solution. Once we collected enough cells, we closed the tube containing the harvested sample, flash-frozen in liquid nitrogen and stored it at −80°C [45]. For each cell types, three independent biological replicates were conducted. Every replicate contained about 1000 cells.

RNA isolation and quantitative real-time (qRT) PCR

Total RNA was extracted from the samples captured by laser microdissection using an SV Total RNA Isolation System (Promega) following the manufacturer's instructions, and complementary DNA (cDNA) was synthesized using a RevertAid H Minus First-Strand cDNA Synthesis Kit (Fermentas). For qRT PCR, probes of 100–150nt length were generated using gene-specific primers (Data S7). The specificity of all primers was confirmed by sequencing the PCR products. The qRT-PCR was performed using TB Green Premix Ex Taq (TaKaRa) in a StepOne Plus Real-Time PCR System (AB Applied Biosystems). The PCR conditions were as follows: initial denaturation at 95 ℃ for 30s, 40 cycles of 95 ℃ for 5s, and 60 ℃ for 30s. Dissociation curves were recorded using 1 cycle of 95 ℃ for 15s, 60 ℃ for 60s, and 95 ℃ for 15s. Relative expression levels were determined by normalizing the PCR threshold cycle number of each gene with that of ACTIN using the $2^{-\Delta Ct}$ method [46]. Each sample was from at least three plants with three biological replicates.

RNA-seq library construction and sequencing

The reverse transcription of RNA and subsequent amplification, library construction, and sequencing were completed using Smart-seq2 (Switching mechanism at 5'end of the RNA transcript) kit (Illumina, USA) [47-48]. At least 30 million 150-basepaired-end reads were sequenced by Illumina Novaseq6000. For sequences mapping, most cells showed at least 76.85% sequence coverage of the *Chirita pumila* genome. Clean reads were obtained from the raw data for each cell type by filtering out adapter sequences and low-quality sequences (quality score<30, length<50bp, and N frequency greater than 10%) using Fastp [49]. Sequences were mapped to the genome and reads were counted by HISAT2 (http://daehwankimlab.github.io/ hisat2/) [50] and StringTie (http://www.ccb.jhu.edu/software/stringtie/) [51], respectively.

Comparative RAN-seq (transcriptome) analysis

The expression level of each gene was normalized by fragments of per kilobase (FPKM of exon per million fragments mapped). Differentially expressed genes were identified using the DESeq2 package (https://github.com/mikelove/DESeq2). Genes with a<0.01 false discovery rate and a>2-fold change were regarded as differentially expressed genes. Gene Ontology (GO) functional classification was performed using the AgriGO with the GO dataset (http://systemsbiology.cau. edu.cn/agriGOv2/). The Kyoto Encyclopedia of Genes and Genomes (KEGG) database (http://www.genome.jp/kegg/) was used to identify the enriched pathways among the DEGs. GO

and KEGG pathways with $P \leq 0.05$ were assigned as significant enrichments. GO enrichment and KEGG enrichment analyses were performed by TBtools [52].

Supplementary References

40. Li ZL. *Plant Sectioning Technique*. Beijing: Science Press, 1978, pp.129-137.
41. Hu SY. Method of preparation of slides used to examine the pollen germination on the stigma and pollen tube growth in the style. *Chin Bull Bot* 1994; **11**: 58-60.
42. Jensen EC. Quantitative analysis of histological staining and fluorescence using ImageJ. *Anat Rec* 2013; **296**: 378-381.
43. Harper J. *Mastering Autodesk 3ds Max 2013*. Indianapolis: John Wiley and Sons, 2012.
44. Laetitia M, Philippe N, Antonio M, Yoshihito S, Carmen C, Jocelyn R. Laser microdissection of tomato fruit cell and tissue types for transcriptome profiling. Nature Protocols 2016; 11:2376-2388.
45. Dong Y, Yang X, Liu J, Wang BH, Liu BL, Wang YZ. Pod shattering resistance associated with domestication is mediated by a NAC gene in soybean. Nature Communications 2014; 5:3352.
46. Yang X, Wang Y, Liu TX, Liu Q, Liu J, Lü TF, Yang RX, Guo FX, Wang YZ. *CYCLOIDEA*-like genes control floral symmetry, floral orientation, and nectar guide patterning. The Plant Cell 2023;10.1093/plcell/koad115
47. Simone P, Omid RF, Asa KB, Gösta W, Sven S, Rickard S. Full-length RNA-seq from single cells using Smart-seq2. Nature Protocols 2014; 9:171-181.
48. Susanne N, Chen G, Julio AB, Marlene Y, Helena S, Ming C, Rickard S, Deng QL, Eva H. Laser capture microscopy coupled with Smart-seq2 for precise spatial transcriptomic profiling. Nature Communications 2016; 7:12139.
49. Chen S, Zhou Y, Chen Y, Gu J. Fastp: an Ultra-Fast All-In-One FASTQ Preprocessor. Bioinformatics 2018; 34: i884-i890.
50. Kim D, Langmead B, Salzberg SL. HISAT: a Fast Spliced Aligner With Low Memory Requirements. Nature Methods 2015; 12:357.
51. Mihaela P, Geo MP, Corina MA, Chang TC, Joshua TM, Steven LS. Stringtie Enables Improved Reconstruction of a Transcriptome from RNA-Seq Reads. Nature Biotechnology, 2015,33:290.
52. Chen CJ, Chen H, Zhang Y, Thomas RH, Frank HM, He YH, Xia R. TBtools: An Integrative Toolkit Developed for Interactive Analyses of Big Biological Data, Molecular Plant, 2020,13:1194-1202.

Supplementary Figures and Legends

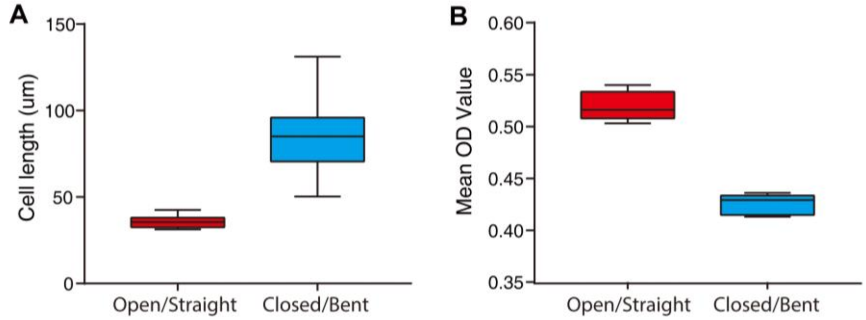

Figure S1. Statistics of contractile cells of open and closed states.
(**A**) Measurements of cell length and cytoplasm density in the contractile cells, showing the average cell length open/straight and closed/bent laminae. (**B**) mean OD values of the open/straight and closed/bent laminae. Values shown (mean ± standard error of the mean) of cell length, and mean optical density were calculated using GraphPad Prism 7.0 software. The difference significance was tested using the LSD test ($P < 0.001$) by Microsoft Excel. Scale bars, 500 µm (A, B), 50 µm (C, D).

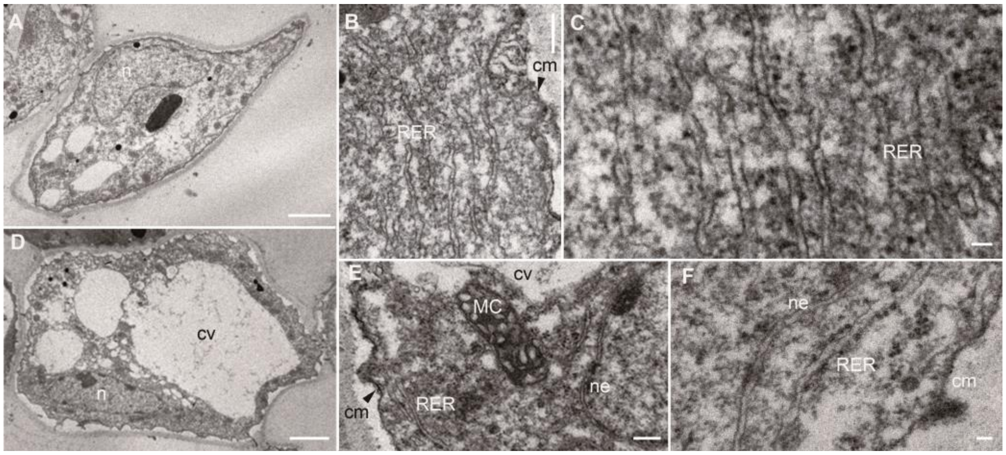

Figure S2. TEM images for comparison of contractile and parenchyma cells.
(**A-C**) TEM images of contractile cells with no obvious vacuolization and the nucleus pressed to the edge (A), abundant rough endoplasmic reticulum within cytoplasmic membrane (cm) distributed throughout the cell (B, C). (**D-F**) TEM images of parenchyma cells giant vacuoles (cv) and the nucleus pressed to the edge (D), a small amount of rough endoplasmic reticulum connected with the nuclear envelope (ne) (E) and distributed only at the cell periphery (F). n, nucleus; MC, mitochondrion. Scale bar, 2µm (A, D), 0.2µm (B, E), 50nm (C, F).

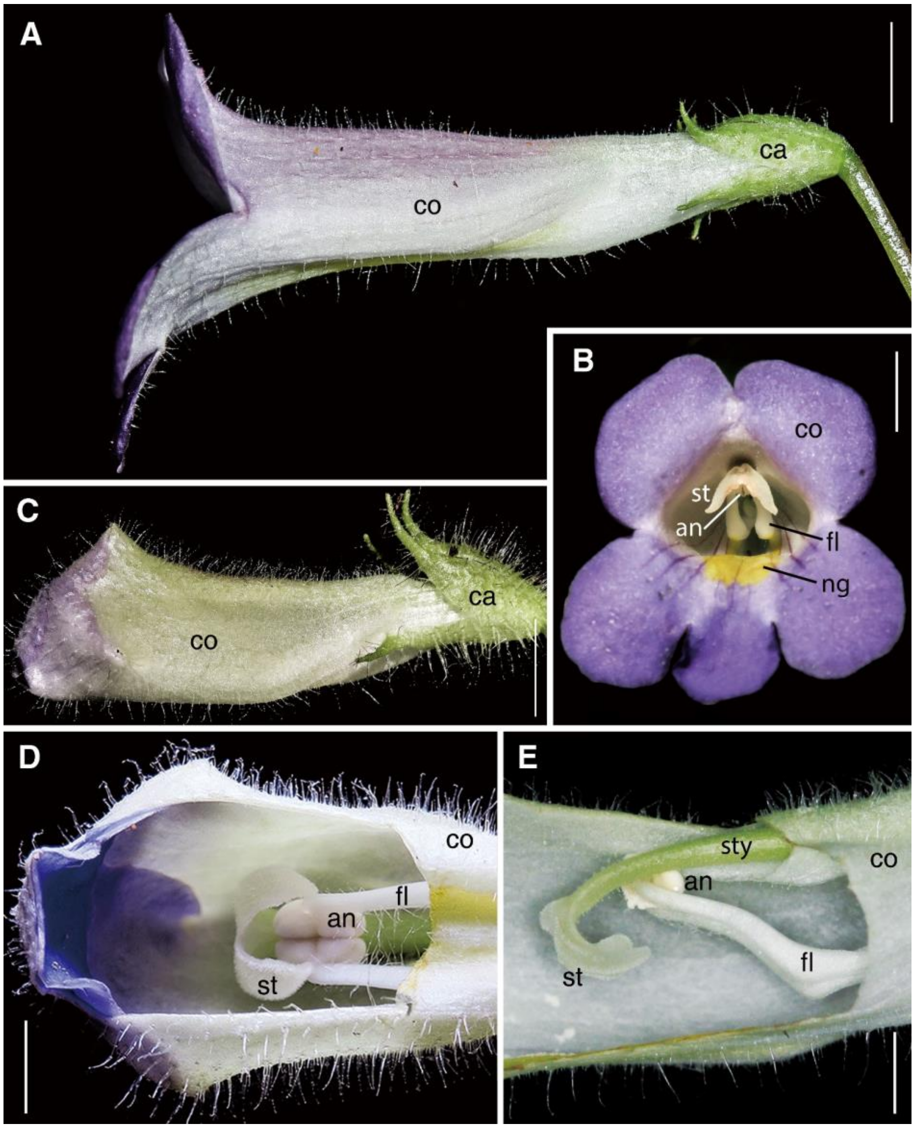

Figure S3. Floral morphology of open flower and closed bud of *Chirita pumila*.
(**A-B**) Lateral **(A)** and front **(B)** view of open flower, showing green calyx (**ca**), light purple bilabiate corolla (**co**), open stigma laminae (**st**), geniculate filaments (**fl**), and coherent anthers (**an**), as well as the light-yellow stripes of the nectar guide (**ng**) on the lower corolla inner surface. (**C-E**) The closed bud with lateral view **(C),** dissected from the ventral (bottom) side **(D)** and lateral side **(E).** Note that the filaments (**fl**) of the two stamens are strongly geniculate at the midpoint and lift up the pair of coherent anthers (**an**) into the position just between the style (**sty**) and abaxial surface of the stigma laminae (**st**), and a large amount of pollen released from precociously mature anthers falls onto the lower inner surface of the corolla tube in the closed bud (**D, E**). Scale bars, 5 mm (A, B, C), 2.5 mm (D, E).

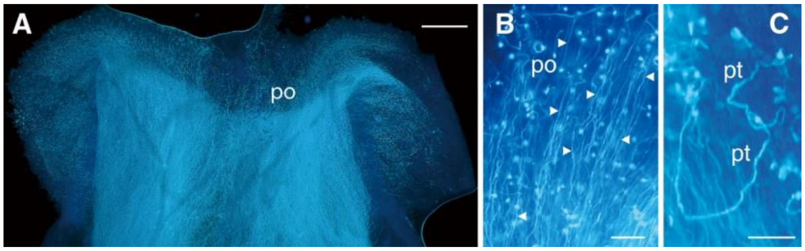

Figure S4. Aniline blue staining of the stigma in the flower bud in *Chirita pumila*.
(**A**) Whole stigma with two laminae, showing abundant pollen grains (**po**) ejected from anthers and germinated on the stigma receptive surface in the closed floral buds. (**B**) Magnitude of an area, showing pollen grains (**po**) germinated with pollen tubes (arrow head). (**C**) A close-up of pollen tubes (**pt**). Scale bars, 500 µm (A), 100 µm (B, C).

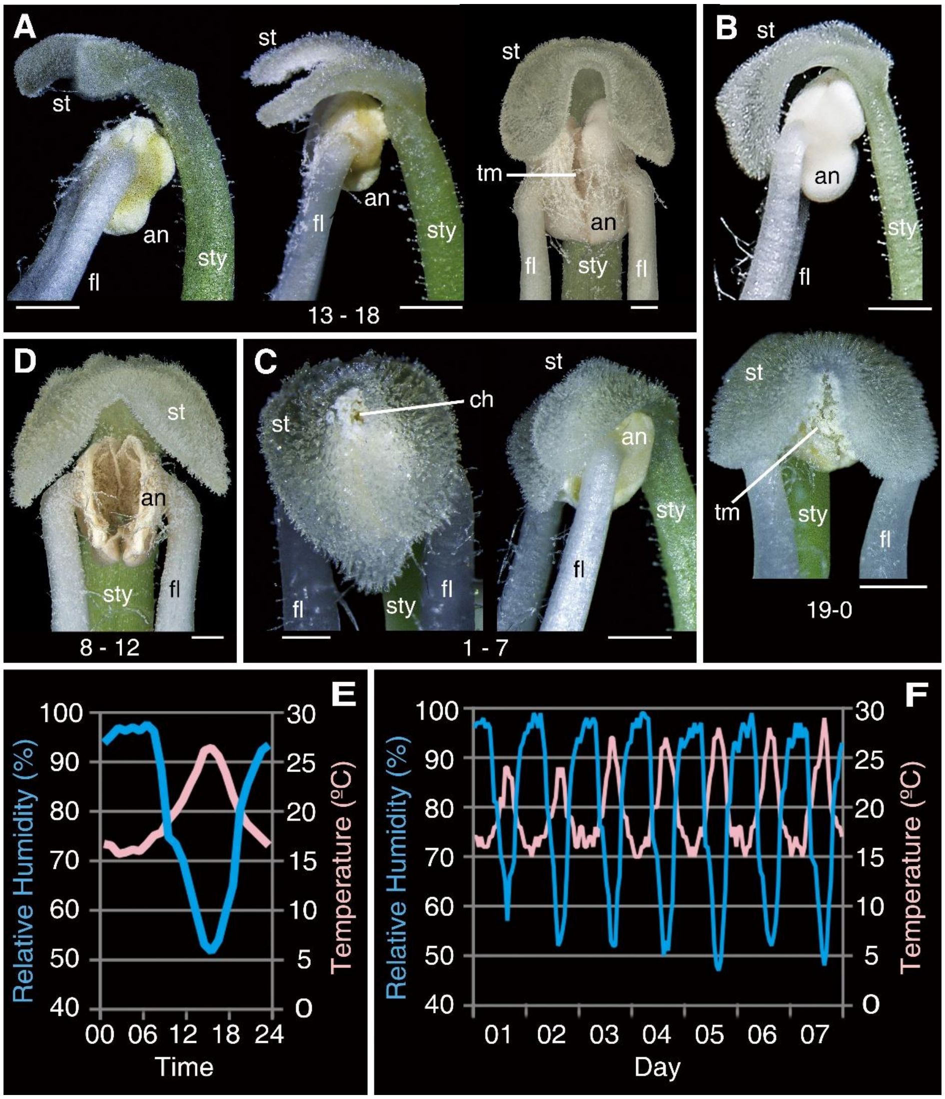

Figure S5. Stigma movement of *Chirita pumila* and relative humidity and temperature variation observed and recorded in the field.

(**A**) Stigma laminae (st) at open state in the afternoon. (**B**) Stigma laminae gradually closing from evening to night. (**C**) Stigma laminae closed from midnight to dawn with lamina tips overlapped, mechanically squeezing anthers (an) against the style (sty). **(D)** Stigma laminae returned to the open state from morning to noon. The direction to the style is upward and the opposite downward. The number below images indicates the time period (Beijing time, UTC+8) corresponding to the state of stigma movement as shown in the images in a circadian cycle (from 13-18, 19-0, 1-7 to 8-12). **Note:** an initial rhombic dehiscent stomium (tm) at the juncture of the face-to-face cohered anthers (an) (**A**) that is extended to full length of the coherent anthers (**B**), and a pollen-channel (ch) formed by stigma movement, making the stomium located just below the inter-lamina hole with movement to closed (**C**). (**E**-**F**) Average daily variation (**E**) and 24-hour continuous real time records of seven-days (**F**) of relative humidity (Blue lines) and temperature (pink lines) at one site in which *C. pumila* grows naturally in Hekou county, Yunnan province, China. Scale bars, 1 mm.

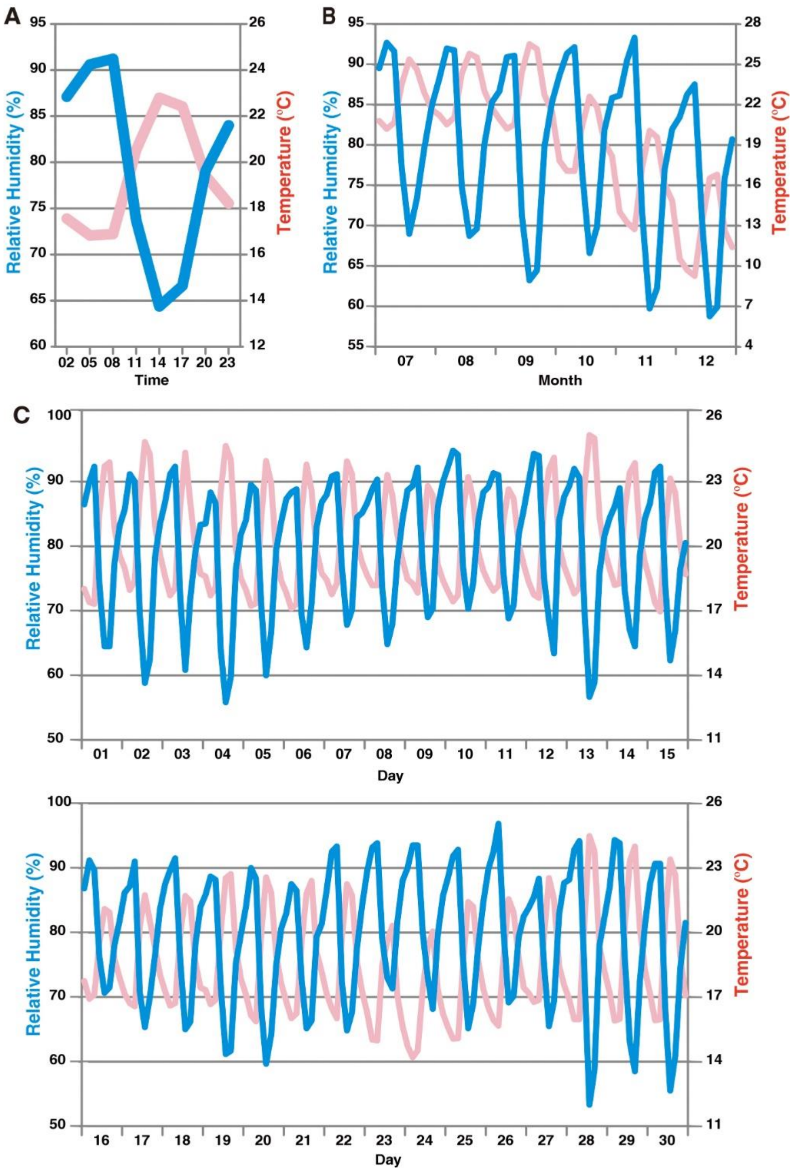

Figure S6. Relative humidity and temperature variation recorded at a weather station. The data on relative humidity and temperature variation were recorded at an official weather station (Mengzi) near the field site of *Chirita pumila* from July 1st to December 31th in 2017. It is located at lat. 23°15'36"N, long. 103°11'24"E and alt. 1313m, Mengzi county, southeastern Yunnan province, Southwestern China. This figure illustrates average daily variation over the six months (**A**), average daily variation in each month **(B)** and average daily variation from 1st to 30th of six months **(C)** of relative humidity (Blue lines) and temperature (pink lines). Chart shows circadian changes with Beijing time, UTC+8 (Data S1).

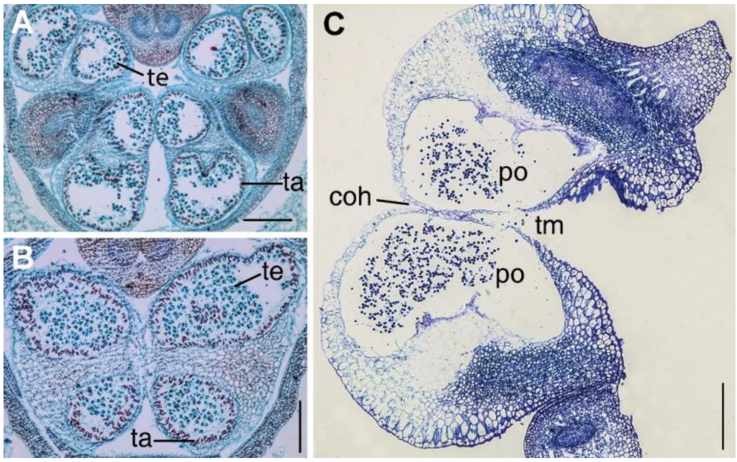

Figure S7. Cross section of anthers of different developmental stages in the bud.
(**A**) Anther with four pollen chambers, showing early pollen tetrad (**te**) and tapetum cell (**ta**). (**B**) Anther with two pollen chambers, showing pollen tetrad (**te**) and tapetum cell (**ta**). (**C**) Precociously mature pollen grains (**po**) with the tapetum disintegrated in the two ripe anthers cohered together, showing coherent portion (**coh**) of two face-to-face anthers, and a dehiscent stomium (**tm**) at the ventral side. Note: pollen grains (**po**) spread out from the dehiscent stomium (**tm**). Scale bars, 200 µm.

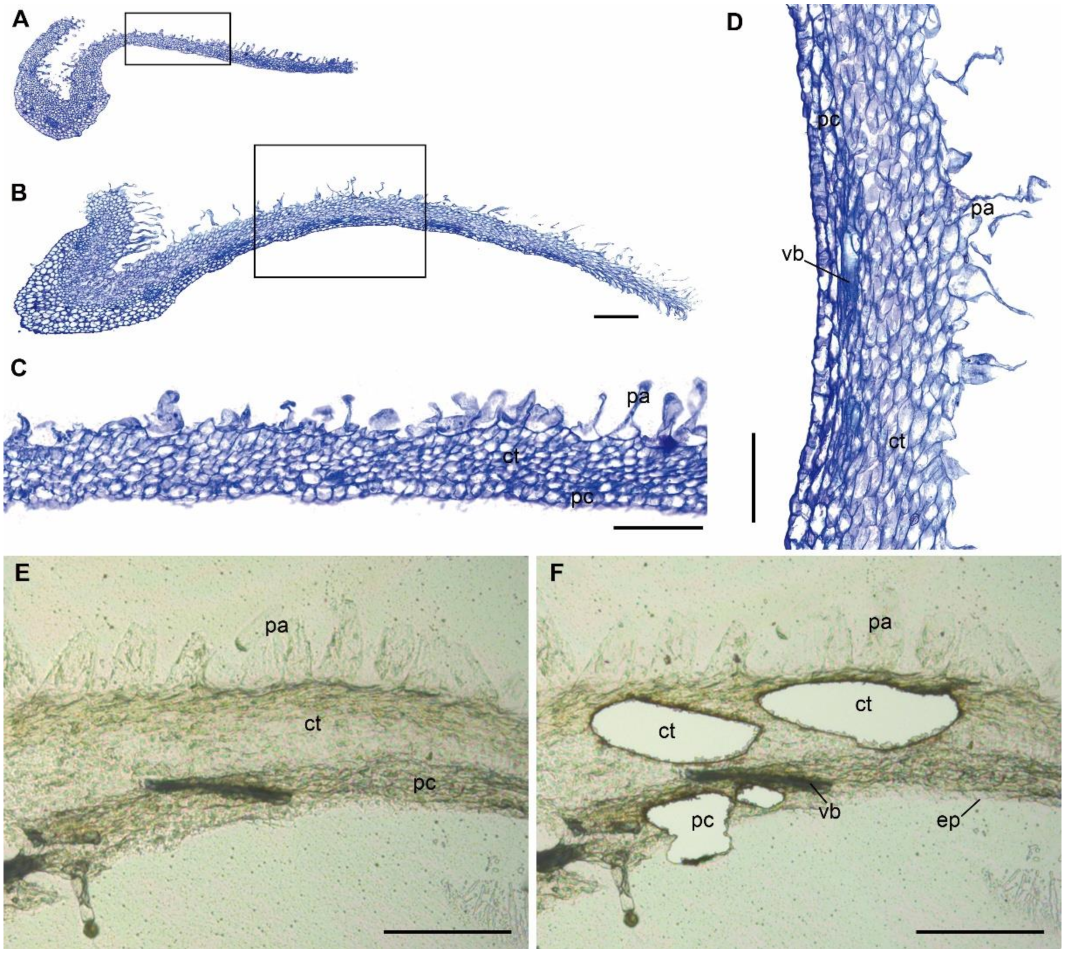

Figure S8. Microscopic section of the stigma of *Chirita pumila* at different developing stages and schematic diagram of laser microdissection.

(**A**, **B**) Longitudinal anatomical sections of the open/straight lamina in young stage (length of flower buds < 0.5cm) (**A**) and mature stage (length of flower buds≈1.5cm) (**B**). (**C**, **D**) magnified view of the area bounded by rectangles in (A) of young stigma (**C**) and in (B) of mature stigma (**D**). (**E**, **F**) Schematic diagram of laser microdissection. Cell morphology before (E) and after (F) laser microdissection. Scale bar, 200μm (A, B, E, F), 100μm (C, D).

Table S1. Relative humidity and temperature data recorded every hour in the field for seven days from 16th to 22th in October 2019 at a site in which *Chirita pumila* occurs naturally.

Date (Oct.) / Time (UTC+8)	16		17		18		19		20		21		22		Average	
	Rel. Hum. (%)	Temp. (℃)	Rel. Hum. (%)	Temp. (℃)	Rel. Hum. (%)	Temp. (℃)	Rel. Hum. (%)	Temp. (℃)	Rel. Hum. (%)	Temp. (℃)	Rel. Hum. (%)	Temp. (℃)	Rel. Hum. (%)	Temp. (℃)	Rel. Hum. (%)	Temp. (℃)
0	96	17	95	16	93	18	97	16	93	17	91	18	92	15	93.9	16.7
1	97	17	97	16	95	18	96	15	94	16	95	18	93	16	95.3	16.6
2	97	16	96	15	97	16	98	15	96	15	97	16	97	17	96.9	15.7
3	97	17	99	16	98	16	96	15	94	16	96	15	94	16	96.3	15.9
4	98	16	98	17	96	17	99	16	97	15	94	16	97	16	97.0	16.1
5	96	16	96	16	97	17	99	16	95	16	97	16	94	15	96.3	16.0
6	97	16	99	17	98	16	98	17	96	17	99	18	96	16	97.6	16.7
7	96	17	95	17	97	18	98	16	97	18	95	18	95	19	96.1	17.6
8	90	17	91	17	89	17	90	18	88	17	89	19	83	20	88.6	17.9
9	81	18	80	18	76	19	74	20	72	19	71	20	70	20	74.9	19.1
10	81	18	77	19	76	19	72	20	72	21	67	21	69	22	73.4	20.0
11	76	19	72	19	71	21	70	20	68	23	66	22	68	24	70.1	21.1
12	70	21	68	20	65	23	64	22	62	24	60	25	62	24	64.4	22.7
13	68	20	59	21	62	24	58	26	52	27	55	26	57	25	58.7	24.1
14	65	24	52	24	53	27	50	26	48	27	53	28	51	27	53.1	26.1
15	57	24	53	23	52	27	53	27	47	28	52	27	48	29	51.7	26.4
16	66	23	55	24	52	25	51	26	49	27	54	27	51	27	54.0	25.6
17	67	23	58	22	63	24	57	25	56	26	57	24	60	23	59.7	23.9
18	69	20	67	19	66	22	61	24	63	24	64	22	65	20	65.0	21.6
19	82	19	81	19	83	20	79	23	80	20	78	19	80	19	80.4	19.9
20	88	18	87	19	88	19	86	20	81	18	83	18	85	19	85.4	18.7
21	92	18	90	18	91	19	91	18	89	17	84	18	86	18	89.0	18.0
22	93	17	92	16	93	17	94	18	93	18	90	17	91	18	92.3	17.3
23	95	16	94	16	93	17	92	17	93	16	94	17	93	17	93.4	16.6

Note: The field site is located at lat. 22°54'6.1''N, long. 104°2'4.23''E and alt. 1366m, Laowang village, Miechang Town, Maguan County, southeastern Yunnan province in Southwestern China. Rel. Hum., relative humidity; Temp., temperature.

Captions for Videos S1 to S6

Video S1.

Water sensitive experiment on the stigmatic laminae in front view. When we put a small quantity of water on the stigma, the two open laminae are immediately closed laterally (and bent upwards toward the style) with the lamina tips partially overlapped, and then the laminae gradually return to their original state with loss of water.

Video S2.

Water sensitive experiment on the stigmatic laminae in lateral view. When we put a small quantity of water on the stigma, the two open laminae are immediately bent upwards toward the style (and closed laterally with the lamina tips partially overlapped), and then the laminae return to their original state with loss of water.

Video S3.

An experiment that simulates natural variation in relative humidity with consistent temperature that causes stigma movement. Following the reduction of the relative humidity from saturation (99%) to 69%, reflecting the natural humidity variation in a day, the stigma laminae move from a closed and strongly bent state to an entirely straight and open state.

Video S4.

Water sensitive experiment on the stigma laminae in lateral view. With water stimulation, the stigma laminae bend rapidly and forcefully, which provide a powerful pressure on the two adherent anthers.

Video S5.

The movement of the stigma laminae with water stimulation and formation of a pollen-channel. With water stimulation, the stigma laminae immediately close and bend, producing a pollen-channel, i.e. the dehiscent stomium of the anthers located just below the inter-lamina hole generated by the stigma movement, and then subsequently the laminae open and straighten gradually with loss of water.

Video S6.

3D model of the stigma movement with subsequent formation of an elaborate complex structure causing self-pollination. In this complex structure, the anther dehiscent stomium is located just below the inter-lamina hole generated by the stigma movement, which forms a pollen-channel. As stigma laminae continuously offer mechanical pressure on the anthers, pollen grains are forcibly ejected from the pollen-channel, falling on the stigmatic receptive surface and causing

self-pollination.

Captions for Data S1 to S7

Data S1.

Measurement data of cell length of contractile cells in the open/straight and closed/bent stigmatic laminae.

Data S2.

Measurement data of mean optical density (OD) value of the contractile cells in the open/straight and closed/bent stigmatic laminae.

Data S3.

Measurement data of the cell length of the contractile cells and parenchyma cells in CryoSEM and CLSM in the open/straight and closed/bent stigmatic laminae.

Data S4.

Multiple of length changes of the contractile cells and parenchyma cells from open/straight to closed/bent stigmatic laminae.

Data S5.

Official record of the relative humidity and temperature at a weather station near the field site of *Chirita pumila* from July 1st to December 31th in 2017. The official weather station, i.e. Mengzi weather station locates at lat. 23°15'36"N, long. 103°11'24"E and alt. 1313m, Mengzi county, southeastern Yunnan province, Southwestern China).

Data S6

GO enrichment analysis of differentially expressed genes (DEGs) between contractile cells (CTs) and parenchyma cells (PCs).

Data S7

The sequence of representative DEGs and the primers used in Real-time PCR.
